# Dragon Fruits as a Reservoir of Natural Polyphenolics with Chemopreventive Properties

**DOI:** 10.3390/molecules26082158

**Published:** 2021-04-09

**Authors:** Paweł Paśko, Agnieszka Galanty, Paweł Zagrodzki, Patraporn Luksirikul, Dinorah Barasch, Alina Nemirovski, Shela Gorinstein

**Affiliations:** 1 Department of Food Chemistry and Nutrition, Jagiellonian University Medical College, 31-008 Kraków, Poland; p.pasko@uj.edu.pl (P.P.); pawel.zagrodzki@uj.edu.pl (P.Z.); 2 Department of Pharmacognosy, Jagiellonian University Medical College, 31-008 Kraków, Poland; agnieszka.galanty@uj.edu.pl; 3 Department of Chemistry, Faculty of Science, Kasetsart University, Bangkok 10900, Thailand; patraporn.l@ku.th; 4 Center for Advanced Studies in Nanotechnology for Chemical, Food and Agricultural Industries, KU Institute for Advanced Studies, Kasetsart University, Bangkok 10900, Thailand; 5 Research Network NANOTEC-KU on Nanocatalysts and Nanomaterials for Sustainable Energy and Environment, Kasetsart University, Bangkok 10900, Thailand; 6 Institute for Drug Research, School of Pharmacy, Faculty of Medicine, The Hebrew University of Jerusalem, Jerusalem 9112001, Israel; dinorah.barasch@mail.huji.ac.il (D.B.); alina.nemirovskai@mail.huji.ac.il (A.N.)

**Keywords:** dragon fruits, cytotoxicity, interaction with drug carrier, antioxidant activity, anti-inflammatory activity

## Abstract

Dragon fruits are a valued source of bioactive compounds with high potential to become a functional food. The aim of the study was to evaluate and compare the chemopreventive potential and chemical composition of fruits harvested in Thailand and Israel. The amount of different compounds in water and methanol extracts and antioxidant activity was investigated. Moreover, cytotoxic activity against cancer and normal cells of skin, prostate, and gastrointestinal origin was performed, accompanied by anti-inflammatory assay based on NO production in RAW 264.7 macrophage model. Additionally, the quenching properties of polyphenols from fruits were determined by the interaction of the main drug carrier in blood human serum (HSA). The chemometric analysis was used to reveal the relationships between the determined parameters. Dragon fruits harvested in Israel revealed higher antioxidant properties and total content of polyphenols and betacyanins when compared to those from Thailand. The examined fruits of both origins showed significant cytotoxic activity toward colon and prostate cancer cells, with no toxic effect on normal cells, but also no anti-inflammatory effect. Moreover, a high binding ability to HSA was observed for water extracts of dragon fruits. All these predestine dragon fruits are the candidates for the attractive and chemopreventive elements of daily diet.

## 1. Introduction

Dragon fruits, obtained from different *Hylocereus* species, are attractive and promising functional fruits, harvested traditionally in Latin America (Colombia, Mexico, Costa Rica, and Nicaragua) and South Asia countries (Vietnam, Thailand). Because of their increasing consumption, new places of harvesting have been considered in different regions of the world such as Australia, Israel, or Reunion Island [1,2]. *Hylocereus* plants are generally hardy, easy to cultivate and quickly produce the fruits [1,2,3], and are resistant to damage during the transport. The fruits may be stored at 8 and 10 °C in good condition and this temperature exerts a protective effect on their quality during storage [4]. All these features provide everyday access to fresh dragon fruits, which meets the recommendations of a diet rich in fruits and vegetables. Except of eating fresh fruits, dragon fruit pulp can be used to make juice, wine, jam, and jelly; seeds may be utilized to extract the oil (about 50% essential fatty acids) or used directly as an ingredient in many food products such as syrup, ice cream, sherbet, candy, yoghurt, and pastries [5].

The biological activity of dragon fruits is attributed to the exceptionally high antioxidant properties, linked with their phenolics and betacyanins content [6]. Because of these compounds, the implementation of dragon fruits to the daily diet may have positive effects on stress-related and inflammation disorders and should be recommended as a prevention in reducing blood pressure, diabetes, alleviating stomach and intestinal problems, or colon cancer [7,8]. Dragon fruits are being considered as a functional food [6], but the information on the amount of bioactive components in the fruits of different origin, available on the market, is still limited. The differences in the content and amount of bioactive compounds in the fruits depend on a sum of elements such as genotype, environmental conditions, maturity, as well as place of harvesting [9,10] and these factors are crucial determinants of the quality of dragon fruits. Thus, to verify if the dragon fruits of different geographical origin are equal in terms of their composition and activity, in this study we performed a comparative analysis of the fruits harvested in Israel (DI) and Thailand (DT). We focused on the major phenolic compounds (flavonoids, phenolic acids, betacyanins), because of their association with beneficial or negative effects on biological activity. The comparison also comprised the antioxidant, cytotoxic, and anti-inflammatory activity of water and methanol extracts of the examined fruits, accompanied by the evaluation of their ability to bind to human serum albumin, the main drug carrier in blood. The chemometric analysis was used to reveal the possible relationships between the determined parameters.

## 2. Results and Discussion

### 2.1. Dragon Fruits Bioactive Compounds and Antioxidant Activities

In the first step of the study we wanted to compare the total content of polyphenolic compounds (polyphenols, flavonoids, flavanols, tannins and betacyanins), followed by the detailed determination of their quantitative profile in the examined dragon fruits. The average content of the bioactive compounds in DI and DT were shown in Table 1. Amongst the examined fruits, DI exhibited a significantly higher level of total polyphenols, flavonoids, flavanols, tannins, and betacyanins in comparison to DT. The average amount of these compounds in dragon fruits is similar to those described previously [11]. The obtained results for DI are in agreement with our previous study on dragon fruits varieties collected in Israel [6]. The results for the phenolic compounds content in dragon fruits are consistent with other authors, who observed higher concentration of these compounds in red than in white pitaya [12,13]. Such strong differences in the content of polyphenols result from the numerous site-specific and environmental factors. Polyphenol concentrations may be affected by harvesting place, maturity stage of fruits, storage conditions, and processing [9,10]. It is well-known that several factors, including the geographical location of orchards, growing and environmental conditions, pedoclimatic parameters, and agronomic methods of cultivation, affect the nutritional quality of different fruits and vegetables [2,14].

The color of dragon fruit flesh comes from betacyanins and betaxanthins content [15]. In this research, significant differences were observed for total betacyanins content among the DI and DT, with DI showing doubled amount in comparison to DT (Table 1). These results are in agreement with the literature data [16], where high content of betacyanin in the peel of red dragon fruit was shown. In our previous study, significant differences in total betacyanins content among the dragon fruits varieties and species were observed, with the highest amount for *H. costaricensis* and the lowest for *H. undatus* [6]. In a recent study [17], betalamic acid, betanin, 2′-O-glucosylbetanin, phyllocactin, hylocerenin, 2′-O-gapiosylbetanin, 2′-O-apiosylphyllocactin were identified in dragon fruits from Israel and Thailand.

Detailed examination of the polyphenolic profile in the tested extracts were performed by HPLC, and the results are presented in Table 1. Flavonoids were found only in the methanol extracts of the tested fruits. Myricetin and rutin were identified in both DI and DT fruits, whereas quercetin was detected only in the former. Myricetin was in significantly higher amounts in DT fruits, whereas the content of rutin was higher in DI fruits, which is in agreement with the literature [18]. The highest level of gallic acid was found in methanol extracts of DI, but in water extracts of DT caffeic and protocatechuic acids were noted. Significant differences in the concentration of gallic acid were observed between methanol and water extracts, which was previously noted [6].

As the presence of polyphenolic compounds is often related to antioxidant activity, the next step of the analysis was directed to its evaluation by four different complementary methods: ABTS, CUPRAC, DPPH, and FRAP (see explanations under Table 2). The results, presented in Table 2, revealed significantly higher activity of DI in comparison to DT. The water extracts showed higher antioxidant activity in comparison to the methanol extracts and these observations were significant in most cases (Table 2). The obtained results were consistent with the literature [6,19], where the higher antioxidant activity was associated with betacyanins content (in red dragon fruits). The antioxidant activity of red dragon fruits may also be related with other non-betalainic phenolics, such as acetylcoumarins or gallic acid [20]. It is in agreement with other authors [8] who suggested that antioxidant activity of dragon fruits is attributed to polyphenols. In this study, significant correlations between DPPH and two pairs of parameters (ABTS and TP; CUPRAC and FRAP), having similar weights, were observed in the partial least square model (PLS). Therefore, DPPH had high correlation weights with all these parameters, particularly with ABTS and TP (Table 3). Some data indicate that antioxidant capacities (ABTS, FRAP) of red dragon fruits were highly correlated with phenolic and flavonoid compounds, but weakly with betacyanins [21], while other authors noted that FRAP and total betacyanins showed a significant strong positive correlation (r = 0.989) and additionally ABTS and total betacyanins (r = 0.718) [22]. The results for dragon fruits antioxidant activity are consistent with those obtained for other functional fruits (durian, mango, avocado, snake fruit, mangosteen and kiwifruit) [10,23].

### 2.2. Cytotoxic Activity

**Figure 1:**
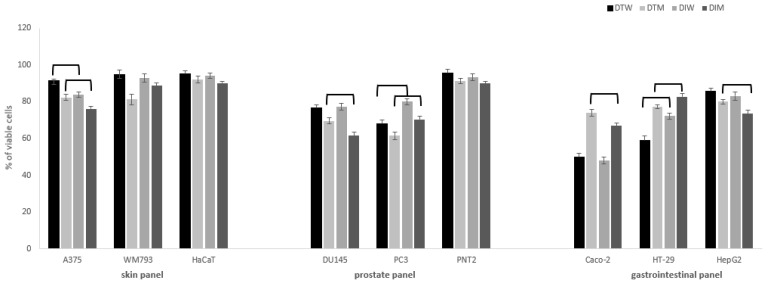
Cytotoxic activity of dragon fruits from Israel (DI) and Thailand (DT) on normal and cancer cells in skin, prostate and gastrointestinal panel. Cells were incubated with fruit water (W) or methanol (M) extracts (0.5 mg/mL). Cell viability was expressed as % of control (untreated) cells (n = 3). Values that are significantly different are combined with upper black line (*p* < 0.05).

In a next part of the study, cytotoxic activity of the tested dragon fruits extracts was evaluated towards the number of human cancer cell lines of different origin and metastatic properties. Cancer cells were grouped in panels (see Cytotoxic activity paragraph in Methods), with corresponding normal cells, to determine not only the cytotoxic potential of the tested extracts, but also their selectivity. The tested DI and DT samples varied in their influence on the examined cell lines, in a dose-dependent manner (0.05–0.5 mg/mL). None of the dragon fruits extracts were toxic for normal cells used in the study (HaCaT, PNT2, HepG2) in the tested concentration range (Figure 1), which indicates their safety and selectivity. 

The significant effects on viability of cancer cells were observed mostly in the case of colon and prostate cancer cells (Figure 1). Melanoma cells were most resistant to the tested extracts when compared to more susceptible prostate and colon cancer cells. In skin panel, only A375 cells were vulnerable to the tested extracts, and DI extracts had higher cytotoxic potential in comparison to DT, for both water and methanol extracts. It is worth noting that cytotoxic activity against Caco-2 and HT-29 colon cancer cells was significantly higher for water than methanol extracts, both for DI and DT. At the concentration of 0.5 mg/mL, DT water extracts presented significantly higher cytotoxic activity against HT-29 cells (59 ± 2% viable cells) in comparison to DI (72 ± 1.5% viable cells), while the opposite effect was observed for Caco-2 cells in the case of methanol extracts (DI 67 ± 1.5% vs. DT 74 ± 2% viable cells). For DU145 prostate cancer cells, higher activity was found for methanol than water extracts, and DI had a stronger influence on cell viability than DT (61.5 ± 1.5 vs. 70 ± 2% viable cells). The cytotoxic effect on highly metastatic PC3 cells of water and methanol DT fruits extracts was significantly stronger (68 ± 2.0; 61.5 ± 2.2% of viable cells, respectively) in comparison to the DI (80 ± 1.7%; 70.4 ± 2% of viable cells, respectively). The cytotoxic activity of different species of dragon fruits extracts was so far rarely evaluated towards different cancer cell lines such as breast, lung, stomach, liver, and colon [24,25,26]. In our previous study, different species and varieties of pitaya fruits harvested in Israel presented similar activity against cancer cells, with the predominant activity for Caco-2 and HT-29 cells [6]. It should be noted that dragon fruits water extracts had significantly higher activity against colon cancer cells than methanol extracts, which may have an implication on its future use in gastrointestinal cancer chemoprevention. On the other hand, methanol extracts revealed a stronger impact on prostate cancer cells when compared to water extracts, which is in agreement with our previous results [6].

**Figure 2:**
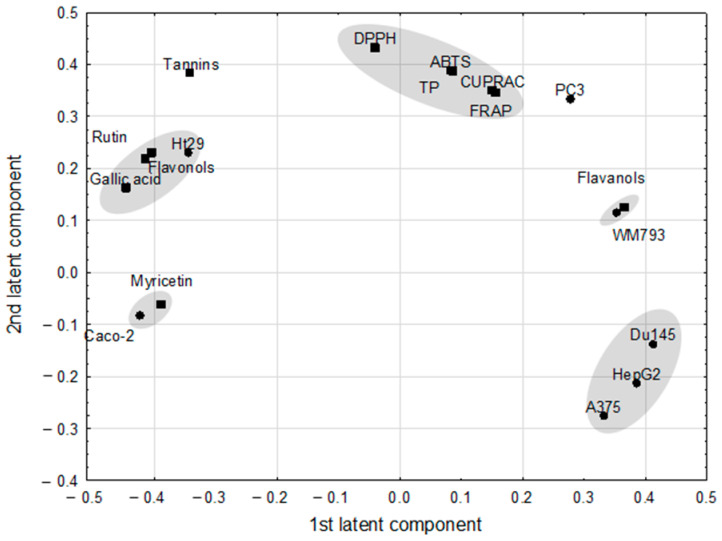
The loadings (coordinates of original parameters, i.e., the weights that combine original parameters with latent components) for first two latent components in the Partial Least Square model. Original parameters with absolute value of their weights higher than 0.3 are considered to be correlated with one another.

The PLS model, which fulfilled cross-validation criteria, showed the correlation structure between the set of predictive parameters with the response ones (Figure 2). The model, with two significant components, explained 96.8% of variance in the predictive parameters and 92.3% of variance in the response parameters, with eigenvalues of 1.89 and 1.98 for the first and second component, respectively. Correlation weights obtained from the PLS model are presented in Table 3. Eleven predictive parameters (ABTS, DPPH, CUPRAC, flavanols, flavonoids, FRAP, gallic acid, myricetin, rutin, tannins, and TP) and seven response parameters (A375, Caco-2, DU145, HepG2, HT-29, PC3, WM793) were included in the PLS model, whereas others were excluded as they were considered non-informative (Figure 2). The first latent component in this model had negative weights predominantly for the gallic acid, flavonoids, rutin and HT-29 (creating an apparent cluster of mutually correlated parameters), as well as for Caco-2 and myricetin (second cluster of parameters), whereas it had high positive weights for response parameters: DU145, HepG2, and A375, and also for WM793 associated with flavanols.

The highest positive correlation weights based on this latent component were revealed between gallic acid and flavonoids and both of them with rutin (Table 3). The PLS model revealed also strong negative correlations between DU145 and Caco-2, and the former parameter with myricetin. Gallic acid correlated negatively with flavanols and WM793.

### 2.3. Nitric Oxide Inhibition

As polyphenolic compounds are known from their anti-inflammatory properties, and the inflammation state often accompanies cancer progression [27], cytotoxic activity of the tested extracts was followed by the determination of their anti-inflammatory potential by means of nitric oxide (NO) assay. Some previously published in vivo studies indicated the anti-inflammatory activity of the extracts of flesh and peels of red pitaya (*Hylocereus polyrhizus*) based on the inhibition of TNF-α and IL-1β [28,29], while no data exist on the inhibitory effect of dragon fruits on NO production. The pre-treatment of the LPS-stimulated macrophages with the tested extracts did not inhibit NO production, when compared to the control cells, treated with the LPS alone. The results suggest that the anti-inflammatory potential of the polyphenolics, present in the tested extracts, may be exerted with a different mechanism than the NO pathway. Thus, this question needs further studies.

### 2.4. Fluorescence Properties of Interaction of Dragon Polyphenols with Human Serum Proteins (HSA)

**Figure 3:**
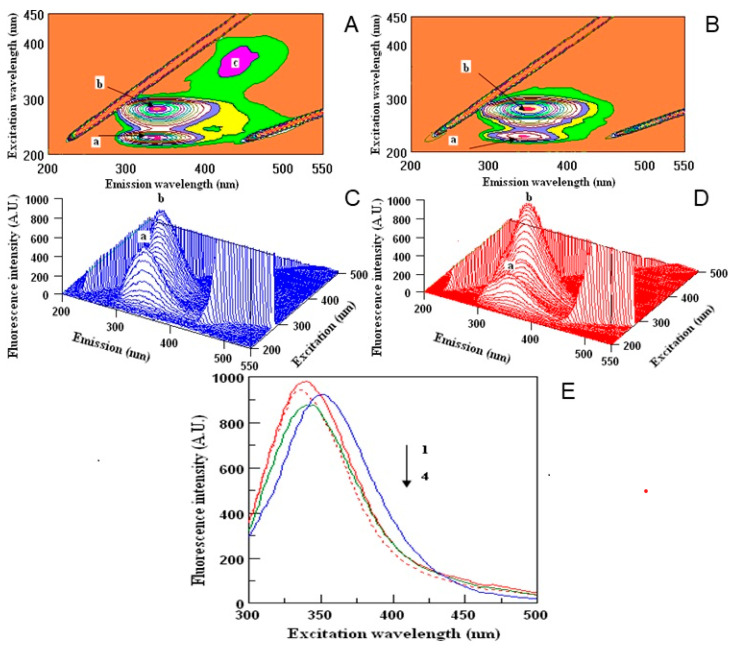
Counter maps (**A**,**B**) and their three-dimensional fluorescence (3D-FL) spectra (**C**,**D**) of interaction of human serum albumin (HSA) with polyphenols water extracts of Thai (**A**,**C**) and Israeli (**B**,**D**) dragon fruits; two-dimensional (2D-FL) fluorescence intensities (FI) from the top (**E**): 1, HSA in buffer; 2, HSA + water; 3, HSA + water extract of Thai; and 4, HSA + water extract of Israeli dragon fruits with λ em (nm) of 340, 336, 352 and 340; FI of 983.18; 945.87; 927.13 and 876.65 arbitral units.

Human serum proteins act as the carriers for different xenobiotics, including polyphenols, enabling their distribution in the organism to obtain the desired effect [30]. Thus, in the last part of the experiment we wanted to examine the ability of the samples to create the complexes with HSA, which may be important in their future use. To determine the binding potential, the fluorescence quenching method was used. The measurements were done for all the samples, but the best results were obtained for water extracts, thus, other images are not presented (Figure 3 and Table 4). 2D-measurements showed that the fluorescence intensities correspond with the amount of polyphenols in two investigated samples of dragon fruits (Figure 3E). The maxima wavelengths in all samples varied from 336 to 352 nm. The fluorescence intensities of the peaks were higher in DT (Figure 3A,C) than in DI (Figure 3B,D) extracts. Peak a was lower in DI nearly twice in comparison with the DT sample, while peak b did not show significant changes during the interaction with HSA. The highest binding properties (55%) were observed for DI, in comparison to DT (15%). Peak c was found only in the DT sample (Figure 3A). The binding properties of the polyphenols in dragon fruits extracts were relatively high, showing the correlation between the antioxidant activity and quenching properties towards HSA.

Most importantly, this interaction was noted for water extracts, which may resemble the form used in a daily diet. This may suggest that dragon fruits, with important health-promoting properties, can be implemented in the human diet equally with other popular fruits.

The different classes of polyphenols found in dragon fruits like flavonols, flavones, flavanones, phenolic acids, or tannins [6,17] have been shown to exhibit many human health benefits due to their antioxidant activity and may have significant influence on first pass metabolism [31]. It was also concluded that evaluation of binding of flavonoids to HSA may be useful for preliminary selection of natural products with the highest anti-proliferative activity [32]. The results of this study also confirmed the correlation between cytotoxic activity of the tested extracts against DU145, HepG2, A375, and WM793 cells and their flavanols content. Moreover, the binding ability with HSA depends on the reactivity and amount of natural phenolic compounds [33,34], which was also proven in our study, with a higher amount of total polyphenols in DI corresponding with its higher binding ability when compared to DT. The present results are in agreement with other authors, where HSA interacted with phenolics in new kiwifruit cultivars [35]. The authors also indicated an important role of hydrogen bond force in binding of polyphenols to HSA.

The 2D-FL measurements were taken at emission wavelengths from 310 to 500 nm and at excitation of 295 nm. For 3D-FL excitation wavelength scan: 200–500 nm. Emission wavelength scans between 200–550 nm. Fluorescence intensity is estimated in arbitral units. Measurements in 3D-FL were done in water extracts with a concentration of 0.17 mg/mL; HSA at 2 M × 10^−5^. The values of peaks a, b are given in Table 4 (for interpretation of the references to color in this figure legend, the reader is referred to the web version of this article).

## 3. Materials and Methods

### 3.1. Chemicals

Trolox (6-hydroxy-2,5,7,8,-tetramethyl-chroman-2-carboxylic acid); 2,2′-azinobis (3 ethylobenzothiazoline-6-sulphonate) (ABTS); FeCl_3_⋅6H_2_O; Folin–Ciocalteu reagent; Tris, tris(hydroxymethy1) aminomethane; lanthanum (III) chloride heptahydrate; CuCl_2_⋅2H_2_O; and 2,9-dimethyl-1,10-phenanthroline (neocuproine, Nc), potassium persulfate, 1,1-diphenyl-2-picrylhydrazyl (DPPH), quercetin, LPS and human serum albumin were from Sigma Chemical Co. (St. Louis, MO, USA). Dimethyl sulfoxide (DMSO), chloroform, HPLC grade acetonitrile, water and formic acid were purchased from Sigma-Aldrich (Seelze, Germany). 2,4,6-Trispyridyl-s-triazine (TPTZ) was purchased from Fluka Chemie (Buchs, Switzerland). Methanol, acetic acid, ammonium hydroxide solution, hydrochloric acid, sodium acetate and sodium carbonate were from Avantor Performance Materials Poland S.A. (Gliwice, Poland).

Standards for HPLC analysis (flavonoids: myricetin, rutin and quercetin; phenolic acids: gallic acid, caffeic acid and protocatechuic acid) were purchased from Fluka Chemie. All reagents were of analytical grade. Distilled water was purchased from Sigma-Aldrich.

### 3.2. Material

**Figure 4:**
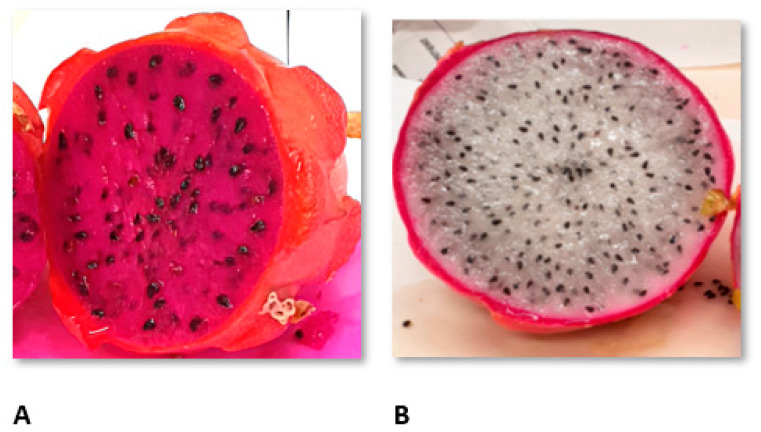
The example of evaluated dragon fruits: (**A**): *H. costaricensis*, with red skin and red flesh harvested in Israel and (**B**): *H. undatus*, with red skin and white flesh harvested in Thailand.

A total of two groups of dragon fruits were included in the study. Fruit samples were collected in accordance with the automatic plan of selecting objects for research from the general population. The plan was based on random rules. The researcher had no subjective influence on the choice of the object. Each item (fruit) had the same chance of being the subject of research. In this way, four objects were selected from a sample containing 30 fruits. The first group of dragon fruits (*Hylocereus costaricensis*), with red skin and red flesh (Figure 4A), was harvested in Israel in 2019 and purchased at the local market (Jerusalem). The second group of fruits (*Hylocereus undatus*), with red skin and white flesh (Figure 4B), was harvested in Thailand in 2019 and purchased at the local market (Bangkok) (Figure 4). For the purpose of this manuscript, the groups were denoted as DI (dragon fruit from Israel) and DT (dragon fruit from Thailand). Immediately after purchase the fruits were peeled and only the flesh was freeze-dried [36] and then extracted. Samples of lyophilized dragon fruits were extracted with methanol or water (3 h, shaking at 19 °C). For cytotoxic assay and nitric oxide determination, the extracts were evaporated under reduced pressure at 50 °C (Heidolph Rotary Evaporator, Schwabach, Germany) and the dry residues were dissolved in DMSO. All extracts for bioactive compounds analysis, antioxidant, and cytotoxic activity measurement were kept at −20° C.

### 3.3. Methods

#### 3.3.1. Determination of Polyphenols, Flavonoids, Flavanols, Tannins and Betacyanins

Polyphenols, flavonoids, flavanols, tannins and betacyanin’s concentration were determined as described previously [6,37]. To evaluate the total amount of polyphenols in the obtained methanol and water extracts of dragon fruits, Folin–Ciocalteu reagent was used. The measurement was performed at 765 nm (with gallic acid as the standard). The results were expressed as mg of gallic acid equivalents (GAE)/g DW. The content of flavonoids (extracted with 5% NaNO_2_, 10% AlCl_3_·6H_2_O and 1 M NaOH) was measured at 510 nm. The total flavanols amount was evaluated using the p-dimethylaminocinnamaldehyde method, and measured at 640 nm. The amount of condensed tannins was determined with 4% methanol vanillin solution, and measured at 500 nm. Catechin was used as a standard for flavonoids, flavanols, and tannins, and the results were expressed as µg or mg catechin equivalents (CE)/g DW. The betacyanin extraction was prepared at 2–4 °C using the procedure described in the literature [38]. Briefly, the dragon fruit flesh was smoothed in a cold mortar with cold distilled water. The homogenates were centrifuged at 4 °C for 15 min. Then, supernatant was shaken with chloroform for 15 min and centrifuged for 30 min at 4 °C. The aqueous phase absorbance was measured at 536 nm, with an amaranthin calibration curve [39]. Betacyanin content was expressed as mg of amaranthin E/100 g DW. Each sample was measured with three replicates.

#### 3.3.2. Determination of Flavonoids and Phenolic Acids

Flavonoids and phenolic acids content were determined as described previously [40], by means of HPLC. Dionex HPLC system with a Photodiode 100 detector was used. The analysis was performed on Hypersil Gold (C-18) column (5 μm, 250 mm × 4.6 mm, Thermo Scientific, Runcorn, UK), with mobile phase 1% formic acid (A) and acetonitrile (B) in a gradient mode 5–60% B over 60 min. The compounds were identified by comparing their retention times and UV spectrum with the standards (see Chemicals section). The quantity of the predominant compounds was determined in relation to the appropriate standard curves (concentrations of 0.0625–1 mg/mL). All the analyses were done in triplicate and the means values were expressed as mg/100 g DW.

#### 3.3.3. Determination of the Antioxidant Activity

Total antioxidant capacities were determined using four complementary tests, namely ABTS (2,2′-azinobis (3 ethylobenzothiazoline-6-sulphonate)), CUPRAC (Cupric Reducing Antioxidant Capacity), DPPH (1,1-Diphenyl-2-picrylhydrazyl), and FRAP (Ferric-Reducing/Antioxidant Power), as previously described in details [6]:

1. ABTS·^+^ was generated by the interaction of ABTS (7 mM) and K_2_S_2_O_8_ (2.45 mM). This solution was diluted with methanol until the absorbance reached 0.7 at 734 nm [41].
2. CUPRAC is based on the use of the copper (II)–neocuproine [Cu (II)–Nc] reagent as the chromogenic oxidizing agent. The tested extracts or standard solution and H_2_O were added to the mixture of Cu (II), Nc, and NH_4_Ac buffer solution. The absorbance at 450 nm was measured against a reagent blank [42].
3. DPPH solution (3.9 mL, 25 mg/L) in methanol was mixed with the tested extracts (0.1 mL). The reaction was monitored at 515 nm until the absorbance was stable [41].
4. FRAP assay measures the ability to reduce ferric-tripyridyltriazine (Fe^3+^-TPTZ) to a ferrous form (Fe^2+^), which absorbs light at 593 nm [43].

#### 3.3.4. Cytotoxic Activity

Human cancer and the corresponding normal cell lines, used in the study, were grouped as follows: skin panel (skin keratinocytes HaCaT, malignant melanoma: A375 and WM793), prostate panel (prostate epithelial cells PNT2, prostate carcinoma: DU145 and PC3), and gastrointestinal panel (hepatocellular carcinoma HepG2, colon adenocarcinoma: Caco-2 and HT-29). Cells were grown at standard conditions (37 °C, 5% CO_2_, relative humidity) and culture media (DMEM/F12 for PNT2, WM 793, HT-29, PC3; DMEM Low Glucose for DU145; DMEM High Glucose for A375, HaCaT; MEM with NEAA for Caco-2), supplemented with 10% fetal bovine serum (FBS) and 1% antibiotics solution (10 000 U penicillin and 10 mg streptomycin/mL). All culture media and supplements were from Sigma-Aldrich. Before the experiment, cells were seeded onto 96-well plates for 24 h (1.5 × 10^4^ cells/well). The culture medium was replaced with fresh medium containing different concentration of the tested extracts (50–500 μg/mL) and incubated for 24 h. Cell viability was measured with the lactate dehydrogenase (LDH) assay (Clontech, Mountain View, CA, USA), as described previously [27]. The absorbance was measured at 490 nm using a Biotek Synergy microplate reader (BioTek Instruments Inc., Winooski, VT, USA). Cell viability was expressed as % of control (untreated cells). Each experiment was done in triplicate.

#### 3.3.5. Inflammation *In Vitro* Model

RAW 264.7 cells were seeded onto 96 multi-well plates (1.5 × 10^5^ cells/well) and pretreated with the tested extracts for 1 h, followed by the addition of 2 ng/mL of LPS to induce the inflammation process, according to the procedure described in [44]. The incubation was continued for the next 24 h.

#### 3.3.6. Nitric Oxide Determination

Griess Reagent Kit was obtained from Promega Corporation (Madison, Winooski, VT, USA), and the nitric oxide evaluation was performed according to the manufacturer’s instructions. The analysis was performed in cell culture supernatant in three replicants, using a Biotek Synergy microplate reader (BioTek Instruments Inc., Winooski, VT, USA) and shown as nitrite concentration (μM).

#### 3.3.7. Fluorometric Measurements

Fluorometric measurements were used for the evaluation of binding properties of dragon fruit extracts to human serum albumin (HSA). Two (2D-FL) and three-dimensional (3D-FL) fluorescence measurements for dragon fruits extracts at a concentration of 0.17 mg/mL were recorded on a model FP-6500, Jasco spectrofluorometer, Tokyo, Japan, equipped with 1.0 cm quartz cells and a thermostatic bath. The 2D-FL measurements were taken at emission wavelengths from 310 to 500 nm and at excitation of 295 nm. The 3D-FL spectra were collected with subsequent scanning emission spectra from 200 to 550 nm at 1.0 nm increments by varying the excitation wavelength from 200 to 500 nm at 10 nm increments. Gallic acid and epicatechin were used as standards [35]. 2.0 × 10^−5^ mol/L HSA; 0.05 mol/L Tris–HCl buffer with 0.1 mol/L NaCl (pH 7.4) were used for the reaction. The binding properties (%) for fluorescence intensity of peaks a and b were calculated by the comparison of the fluorescence intensities between initial HSA and the final one after interaction with the investigated dragon fruits extracts.

#### 3.3.8. Statistical Analysis

Descriptive statistics were calculated for all parameters. The differences between two extracts were tested using t-Student test, applied when results for the two others were below LOD, while ANOVA with post hoc Tukey test were used to check the differences between all four extracts. A probability level of *p* < 0.05 was considered to be statistically significant. Partial Least Square model (PLS) was used in order to describe the correlation structure between parameters. The parameters with large weights (>0.3) in PLS model were assumed to be correlated with one another. In order to express the strength of bivariate associations, for the pairs of correlated parameters the algebraic products of their corresponding weights and cosine of corresponding angle were calculated (these coefficients are called the correlation weights). The corresponding angle means the angle determined by two lines connecting the origin with coordinates of both parameters on the PLS weights plot. Statistical analyses were carried out using packages STATISTICA v.13 (Statsoft, Tulsa, OK, USA; descriptive statistics, diagrams), Graph‑Pad Prism v.3.02 (GraphPad Software, San Diego, California, United States; Student *t*-test, ANOVA), SIMCA-P v.9 (Umetrics, Umeå, Sweden; PLS method). The software delivered by MP System Co. (Chrzanów, Poland) was used to calculate correlation weights for pairs of parameters in PLS.

## 4. Conclusions

The results indicate that dragon fruits harvested in Israel have higher antioxidant potential than the fruits from Thailand, which confirms that the place of harvesting and the variety of the fruits may be of significance for their biological activity. However, the question of the interchangeable use of the fruits of different geographical origin and their equality in terms of the composition and health-beneficial effects is still open and requires further studies. The examined dragon fruits, with their high antioxidant properties and cytotoxic potential to colon cancer cells and selectivity to normal cells may become an important chemopreventive element of daily diet. Our results also demonstrate that dragon fruits polyphenols have high ability to HSA binding, thus providing their appropriate distribution in the organism and obtaining the desired beneficial effect. This feature is a prompt for future direction in further in vivo studies.

## Figures and Tables

**Table 1:** Chemical composition of dragon fruits harvested in Israel (DI) and Thailand (DT) (mean value ± SD).

Parameter	Dragon Fruits from Israel (DI)		Dragon Fruits from Thailand (DT)	
	Methanol (DIM)	Water (DIW)	Methanol (DTM)	Water (DTW)
	**Total Bioactive Compounds**			
Total Polyphenols [mg GAE/g DW]	7.78 ± 0.24 ^a1c1^	9.11 ± 0.35 ^a1d1^	2.51 ± 0.10 ^b1c1^	4.52 ± 0.19 ^b1d1^
Flavanols [µg CE/g DW]	60.40 ± 4.42 ^a1c3^	111.75 ± 4.35 ^a1d1^	46.94 ± 5.00 ^b1c3^	81.23 ± 4.43 ^b1d1^
Flavonoids [mg CE/g DW]	1.57 ± 0.10	−	0.71 ± 0.14	−
Tannins [mg CE/g DW]	3.07 ± 0.12	2.42 ± 0.26	2.29 ± 0.22	1.37 ± 0.25
Total Betacyanins [mg/100 g DW]	9.30 ± 1.53 ^a1b2^	2.56 ± 0.25 ^a1c3^	5.33 ± 0.12 ^b2^	4.99 ± 0.21 ^c3^
	**Selected Flavonoids [mg/100 g DW]**			
Myricetin	8.71 ± 0.79 ^a1^	Nd	17.95 ± 0.32 ^a1^	Nd
Rutin	3.67 ± 0.28 ^a1^	Nd	1.46 ± 0.13 ^a1^	Nd
Quercetin	Nd	Nd	6.37 ± 0.14	Nd
	**Selected Phenolic Acids [mg/100 g DW]**			
Gallic acid	34.26 ± 1.75 ^a1c1^	1.92 ± 0.59 ^a1^	23.86 ± 0.70 ^b1c1^	1.32 ± 0.26 ^b1^
Caffeic acid	Nd	Nd	Nd	1.25 ± 0.27
Protocatechuic acid	Nd	Nd	Nd	0.57 ± 0.16

Means and standard deviations for bioactive compounds (n = 3) studied in dragon fruits (results marked with the same letter in upper index within each row differ significantly; levels of significance 1: *p* < 0.001, 2: *p* < 0.01, 3: *p* < 0.05). Abbreviations: CE—catechin equivalent; GAE—gallic acid equivalent.

**Table 2:** Antioxidant activity of dragon fruits harvested in Israel (DI) and Thailand (DT) (mean value ± SD).

Parameter	Dragon Fruits from Israel (DI)		Dragon Fruits from Thailand (DT)	
	Methanol (DIM)	Water (DIW)	Methanol (DTM)	Water (DTW)
ABTS [µM Trolox/gDW]	21.85 ± 0.73 ^a2b1^	24.49 ± 0.94 ^a2c1^	15.82 ± 0.42 ^b1^	17.53 ± 0.58 ^c1^
CUPRAC [µM Trolox/gDW]	22.28 ± 0.70 ^a1c1^	26.57 ± 1.15 ^a1d1^	16.14 ± 0.61 ^b2c1^	18.98 ± 0.50 ^b2d1^
DPPH [µM Trolox/gDW]	13.65 ± 0.37 ^a3b1^	14.69 ± 0.46 ^a3c1^	5.11 ± 0.28 ^b1^	5.04 ± 0.18 ^c1^
FRAP [µM Trolox/gDW]	9.06 ± 0.57 ^a3b2^	10.76 ± 0.76 ^a3c1^	6.77 ± 0.13 ^b2^	7.86 ± 0.35 ^c1^

Means and standard deviations for antioxidant capacity (n = 3) studied in dragon fruits (results marked with the same letter in upper index within each row differ significantly; levels of significance 1: *p* < 0.001, 2: *p* < 0.01, 3: *p* < 0.05). Abbreviations: ABTS-2, 2-Azino-bis (3-ethyl-benzothiazoline-6-sulfonic acid) diammonium salt; CUPRAC-Cupric reducing antioxidant capacity; DPPH-1,1-Diphenyl-2-picrylhydrazyl; FRAP-Ferric-reducing/antioxidant power.

**Table 3:** Correlation weights for the pairs of parameters based on PLS model (only correlation weights with absolute values higher than 0.149 were shown).

Pairs of Correlated Parameters		Correlation Weights
Gallic acid	Flavonoids	0.184
Gallic acid	Rutin	0.178
Rutin	Flavonoids	0.168
DPPH	ABTS	0.167
DPPH	TP	0.167
Myricetin	Caco-2	0.165
Gallic acid	Caco-2	0.162
HepG2	DU145	0.157
Myricetin	Gallic acid	0.152
TP	ABTS	0.151
Gallic acid	HT-29	0.150
Flavanols	Flavonoids	–0.150
HepG2	Caco-2	–0.156
Gallic acid	WM793	–0.158
Myricetin	DU145	–0.159
Gallic acid	Flavanols	–0.163
DU145	Caco-2	–0.173

**Table 4:** Fluorescence results of 3D-measurements of water dragon extracts (DT and DI) in interaction with human serum albumin (HSA).

Sample	Peak a		Peak b		Peak c	
	λ_ex_/λ_em (nm/nm)_	Int F_0_	λ_ex_/λ_em (nm/nm)_	Int F_0_	λ_ex_/λ_em (nm/nm)_	Int F_0_
**HSA + Water**	228/347	744.10	280/350	853.41	–	–
**HSA + DT**	230/345	634.15	281/341	850.53	366/439	132.45
**HSA + DI**	228/345	335.22	280/349	871.70	–	–

Abbreviations: Int F_o_- fluorescence intensity; A.U. -arbitral units.

## Data Availability

Data available on request.

## Sample Availability

Not available.
